# Adsorption of Organic Pollutants from Wastewater Using Chitosan-Based Adsorbents

**DOI:** 10.3390/polym17040502

**Published:** 2025-02-14

**Authors:** Ernestine Atangana, Timothy Oladiran Ajiboye, Abolaji Abiodun Mafolasire, Soumya Ghosh, Bello Hakeem

**Affiliations:** 1 Centre for Environmental Management, University of the Free State, Bloemfontein 9300, South Africa; 2 Department of Chemistry, University of the Free State, Bloemfontein 9300, South Africa; 3 Industrial Unit, Chemistry Department, University of Ibadan, Ibadan 200001, Nigeria; abolajiabiodun007@yahoo.com; 4 Natural and Medical Sciences Research Center, University of Nizwa, Nizwa 616, Oman; s.ghosh@unizwa.edu.om; 5 Science Laboratory Department, Federal College of Fishery and Marine Technology, Lagos 106104, Nigeria; hakeembello34@gmail.com

**Keywords:** adsorption, organic pollutants, chitosan, environmental remediation

## Abstract

Among the naturally occurring polysaccharides, chitosan is the second-most abundant polysaccharide. It is obtained from chitin through a process known as deacetylation. It is biodegradable, biocompatible, and non-toxic, which made it suitable for various environmental applications. In the present review, the structure, properties, and characteristics of chitosan were discussed. In addition, the modified forms of chitosan (including cross-linked, nanoparticles, functionalized, and grafted forms of chitosan) were enumerated. The applications of these modified forms of chitosan in the adsorption of organic pollutants (such as antibiotics, dyes, pesticides, microplastics, polyaromatic hydrocarbons, parabens, and polychlorobiphenyls) are comprehensively reviewed. Furthermore, the mechanism of adsorption, adsorption isotherm (Langmuir and Freundlich), and the kinetic models are highlighted. Finally, the economic viability assessment and environmental impact of processing tons of shrimp shells into chitosan annually were discussed.

## 1. Introduction

A clean water supply is an essential requirement for a suitable, healthy community. It is a source of potable water used for drinking, irrigation, and domestic purposes. It supports the growth of aquatics, thereby providing valuable food supplements. Although these natural sources of life are essential to almost all living beings, they are subjected to pollution [1]. The problems associated with acid mine drainage effluents produced from coal mines, abattoir effluents, and wastewater discharge into rivers and streams are well-known; yet, finding methods to treat various waste types is not always easy [2,3]. Typical pollutants in effluent wastewater include phenol, dyes, and persistent organic pollutants [4,5]. Due to the impacts of these pollutants, there is an increasing demand for efficient removal techniques. Examples of the techniques that have been used are adsorption, bioremediation, reverse osmosis, ion exchange, sedimentation, and filtration. Out of these methods of removal, adsorption stands out and is widely used because of minimal cost and ease of operation [2,6]. The limitation on the use of activated carbon is the relatively high cost and the problem of secondary pollution. As a result, chitosan has been considered as an adsorbent for the adsorption of these pollutants from wastewater. The presence of organic pollution of water is a serious problem for the environment and public health. Because of these pollutants’ toxicity, bioaccumulation, and persistence, ecosystems and human populations are seriously at risk [7,8,9,10].

Both naturally occurring processes and human activity, such as mining, industrial discharge, agricultural runoff, and inappropriate waste management, can introduce these pollutants into aquatic systems [11,12]. The use of pesticides and fertilizers, which frequently contain trace levels of metals like cadmium and arsenic that can leak into groundwater or wash off into surface waters, is another way that agricultural practices contribute [3,11]. Both people and wildlife are in serious health danger from these pollutants.

Organic pollutants pose a serious threat to water systems. These include herbicides, pesticides, medicines, and industrial chemicals, including polychlorinated biphenyls (PCBs) and polycyclic aromatic hydrocarbons (PAHs). These substances are distinguished by their capacity for long-distance transit, environmental persistence, and bioaccumulation in living beings [13,14]. Agricultural runoff serves as a major reservoir for organic pollutants, as weed and pest management measures often involve the application of herbicides and pesticides. These substances pose a concern to aquatic life as well as human health because they can seep into groundwater or be transferred into surface waters by runoff [15,16]. Chemicals like PAHs and PCBs, which are by-products of processes like combustion and chemical manufacture, are released because of industrial operations [17,18]. Personal care and pharmaceutical products (PPCPs) are a growing category of organic contaminants that are mostly introduced into water bodies via home wastewater. Because conventional wastewater treatment plants are frequently ill-equipped to eliminate these materials, the aquatic environment continues to contain them [19]. Organic pollutants can harm both the environment and human health in several ways. Herbicides and pesticides have been connected to reproductive problems in both people and wildlife, endocrine disruption, and cancer. For example, exposure to the common herbicide atrazine has been linked to endocrine disruption in amphibians, which has resulted in population decreases [20]. Human liver damage and developmental issues have been linked to the well-known carcinogens (PCBs and PAHs) [21]. Organic contaminants in aquatic environments can interfere with aquatic organisms’ ability to reproduce and develop, which can result in population and biodiversity reductions. In addition to disrupting food webs and ecosystem processes, these contaminants’ persistence and bioaccumulation [22].

A comprehensive strategy incorporating technological advancements, public awareness campaigns, and regulatory actions is needed to address the contamination of water caused by heavy metals and organic contaminants. To restrict the amount of organic pollutants and heavy metals that are released into the environment, governments and international organizations have set rules and laws. Examples of such laws designed to preserve water quality are the Clean Water Act of the United States and the Water Framework Directive of the European Union [23]. Technological developments in water treatment, including bioremediation, adsorption strategies, and sophisticated oxidation processes, present viable ways to eliminate organic and heavy metal contaminants from tainted water [24]. Adsorbents such as activated carbon and biochar, for instance, are efficient in eliminating a variety of pollutants, such as organic pollutants and heavy metals [25].

Similarly, chitosan is a linear polysaccharide that is commonly employed as an adsorbent. It is made up of randomly distributed units of *N*-acetyl-D-glucosamine and β-(1→4)-linked D-glucosamine [26,27] (Benettayeb, Ahamadi et al., 2024; Benettayeb et al., 2023). The solubility, biocompatibility, and bioactivity of chitosan are all influenced by its varying degree of deacetylation (DD). Higher DD chitosan generally offers better antibacterial and adsorption qualities, is more soluble in acidic conditions, and can be used for water treatment [28] (Suyambulingam et al., 2023). Because its amino groups have been protonated, it becomes cationic in acidic solutions, making it easier for it to interact with impurities that are negatively charged. The adsorption of heavy metals, dyes, and other contaminants from aqueous solutions is made easier by this characteristic [29] (Bhatnagar & Sillanpää, 2009). For instance, a prior study showed the considerable reduction of lead, copper, and mercury concentrations in water through the adsorption of chitosan films and beads [28] (Suyambulingam et al., 2023). Therefore, this review discusses, analyses, and summarizes important empirical findings on the use of chitosan as a natural adsorbent for removing organic pollutants and heavy metals from the environment. It is based on the literature that was retrieved from Google Scholar, Publisher Medline, and a number of recent studies.

## 2. Mechanisms of Adsorption Processes

The mechanism of adsorption requires that the ions or molecules of liquid or gaseous phase remain on the surface of the solid through mass transfer. While these liquid and gaseous molecules are the adsorbate, the solid materials used for their adsorption are called adsorbents [30] (Mahmood Aljamali & Obaid Alfatlawi, 2021). The process is a surface phenomenon, which implies that the adsorbate molecules will not penetrate the bulk of the adsorbent material. It depends on the equilibrium processes for separating contaminants from wastewater. Compared to other remediation methods, adsorption is easy to use; the design is simple and cheap. In addition, the formation of hazardous intermediate products is not possible, and it is not sensitive to toxic species in the wastewater [31,32].

**Figure 1:**
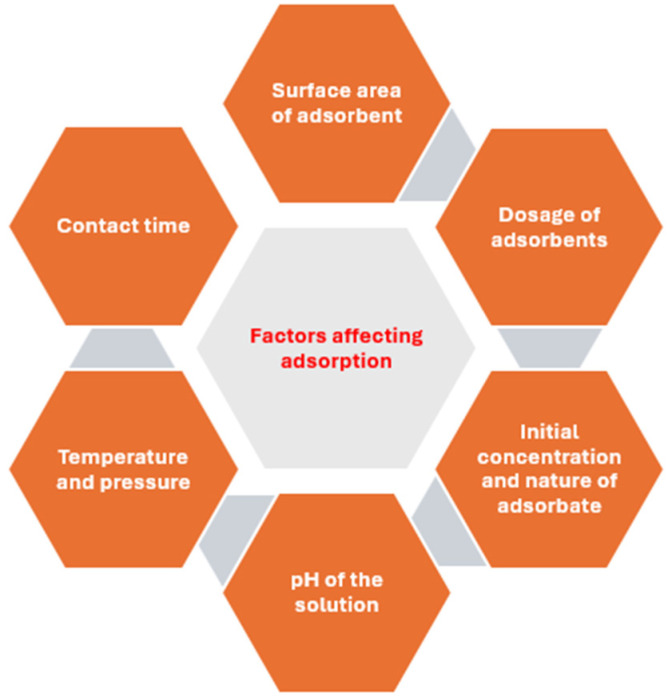
Factors affecting the rate of adsorption.

In adsorption, the adsorbents are generally made in such a way that there will be porosity in their internal structure through which the adsorbate molecules or ions are retained [33]. The adsorption is favoured by the affinity of the adsorbate for the adsorbent, and the adsorbate is retained on the adsorbent by the attractive forces and surface energy between the adsorbate and adsorbent. Based on how the adsorbate binds to the adsorbents, the adsorption process can be classified into physisorption and chemisorption. The differences between these two processes are listed in Table 1. Generally, the rate of adsorption is a function of the surface area per unit mass of the adsorbent. Other factors that affect the efficiency of adsorption are shown in Figure 1. After the adsorption has taken place, the process of removing the adsorbate from the adsorbent is called desorption, which is the opposite of adsorption.

### 2.1. Freundlich and Langmuir Adsorption Isotherms

The significance of adsorption isotherms is for monitoring of adsorption capacity as determined by the experimental parameters. They always enhance the comparative study of various adsorbents [34]. From the findings carried out so far, it can be extrapolated that most adsorption processes involving chitosan-based materials always conform with either Freundlich or Langmuir isotherms.

#### 2.1.1. Langmuir Adsorption Isotherm

The Langmuir model gives the rudiment of what is happening between the adsorbate and adsorbents. It has been applied in different fields, including but not limited to material sciences, biological sciences, and chemical sciences [35]. It describes the equilibrium existing between the adsorbent and adsorbate based on monolayer assumptions at a point where the unit pressure is reached [36].The model also assumes that the desorption rate from a surface varies directly with the fraction covered by the adsorbate (*θ*). It also assumes that the rates of adsorption and desorption are the same at equilibrium for a specific binding site. Another assumption is that all the sites of adsorption are similar, and there is no interaction between the adsorbed molecules on the surface of the adsorbent. It has primarily been used for chemisorption, but its application has been extended to binary systems [37,38]. The linearized expression of Langmuir is as follows:

(1) $$\frac{C_{e}}{q_{m}}=\frac{1}{K_{L}q_{max}}+\frac{C_{e}}{q_{max}}$$

where *K_L_* is the Langmuir constant, *q_m_* is the quantity of adsorbed molecules in mg/g, *q_max_* is the adsorption capacity in mg/g, and *C_e_* is the equilibrium molecules concentration in milligrams per litre.

#### 2.1.2. Freundlich Adsorption Isotherm Model

Unlike Langmuir, which is used for unit layer adsorption, Freundlich is considered when the heterogenous molecules are adsorbed on the surface of the adsorbent. It is simply for describing multilayer adsorption taking place on the heterogenous surface [37,38]. This isotherm can be represented as follows:

(2) $$\log q_{m}=\log K_{f}+\frac{1}{n}\log C_{e}$$

where *q_m_* is the quantity of adsorbed molecule in mg/g, *C_e_* is the equilibrium molecules concentration in milligrams per litre, 1/*n* is the intensity of adsorption, and *K_F_* is the Freundlich constant.

It should be noted that the value of *n* is always more than one, which implies that the amount of gas adsorbed does not increase rapidly with increasing pressure. When the pressure is low, there is direct variation between the adsorption and the amount adsorbed, but this does not hold at a high pressure [39,40]. From the Freundlich expression, it shows that the site of energy distribution for the adsorption site does not display true exponential. However, at low solute concentration, there is semi-exponential energy distribution, and this conforms to the Freundlich isotherm expression [40,41].

### 2.2. Kinetic Models

The study of the kinetics of the adsorption of pollutants onto chitosan-based materials is very important as it helps in the optimization and modelling of the adsorption parameters, process cost, and technology [34]. This would further help in the investigation of adsorption capacity. However, adsorption isotherms help in understanding the kind of interaction formed between the adsorbent and adsorbate during the process, giving insight into the optimum utilization of an adsorbent [42]. Carolin et al. (2009) reported the kinetics for the adsorption of TC onto chitosan and found out that the kinetics followed the pseudo-nth-order process, which was determined by many factors such as the concentration of TC, time of adsorption, the ratio between active adsorption sites, and the number of adsorbate molecules. This agrees with the report by Morais et al. (2008), where it was discovered that the adsorption of methyl orange on chitosan obeyed pseudo-nth-order and fitted well with Freundlich–Langmuir isotherms [43].

Most adsorption processes using chitosan-based materials had been reported to follow pseudo-second-order kinetics and also complied with Langmuir and Freundlich isotherms. An adsorbent CS@TDI@EDTA@γ-AlO(OH) prepared from chitosan, toluene diisocyanate (TDI), ethylene diamine tetra acetic acid (EDTA), and Al(NO_3_)_3_⋅9H_2_O/NaOH was used for the removal of TC and diazinon. The equilibrium results at the optimal temperature, pH, adsorbent dosage, and adsorption time revealed that the adsorption of TC and diazinon fitted well with Langmuir and Freundlich isotherms, respectively. In the kinetics of the adsorption process, the correlation factor (R^2^) for the pseudo-second-order model was 0.9986 and 0.9988 for diazon and tetracycline, respectively [42].

Expressions for pseudo-first-order and pseudo-second-order are represented by Equations (3) and (4), respectively.

(3) $$\log\left(q_{e}-q_{t}\right)=\log\;q_{e}-\left(\frac{k_{1}}{2.303}\right)t$$

(4) $$\frac{t}{q_{t}}=\frac{1}{k_{2}q_{e}^{2}}+\left(\frac{1}{q_{e}}\right)t$$

where

*q_e_* (mg.g^−1^) = adsorption capacity at equilibrium.
*q_t_* (mg.g^−1^) = adsorption capacity at time t.
*k*_1_ (s^−1^) = rate constant for first-order adsorption.
*k*_2_ (mol L^−1^ s^−1^) = rate constant for second-order adsorption.
*t* (min) = time of adsorption.

The Weber–Morris kinetic model is equally relevant as the pseudo-first- and second-order models in investigating the adsorption capacity of the adsorbent. In single and multicomponent adsorption of three antibiotics (amoxicillin (AMX), ciprofloxacin (CIP), and sulphamethoxazole (SMX)) using chitosan-carbon nanotube hydrogel beads, at pH 7, the single kinetic experimental data obtained for AMX, CIP, and SMX fitted with the nonlinear pseudo-first-order model. However, the Weber–Morris kinetic model revealed that the rate of adsorption of the three antibiotics was reduced as a result of multiple processes [44]. The Weber–Morris kinetic model always shows the intraparticle diffusion process by deducing the value of the Weber–Morris rate constant when the q_t_ is plotted against time t. The plot of the adsorption process obeying the Weber–Morris model always passes through the origin (0, 0) if the process is controlled by intraparticle diffusion. Moreover, non-Weber–Morris adsorption processes are governed by multiple adsorption processes. Hence, the rate-determining step of the adsorption process can easily be ascertained [45]. Mathematical expression is shown in Equation (5).

(5) $$q_{t}={\mathrm{kt}}^{0.5}$$

where

*q_t_* (mg.g^−1^) = adsorption capacity at time t.
*k* (min^−1^) = Weber–Morris rate constant.
*t* (min) = adsorption time.

Table 2 presents the kinetic models and isotherm of a few examples of adsorption processes where chitosan-based materials were employed, in their various forms.

## 3. Structure and Characteristics of Chitosan and Its Derivatives

### 3.1. An Outline of the Chemical Structure of Chitosan

A biopolymer produced from chitin, chitosan has extraordinary properties that help its extensive variety of use in enterprises like food technology, medicine, environmental management, and agriculture. The chemical structure of chitosan is analysed in this review, along with its atomic makeup, sub-atomic weight, properties of hydrogen bonds, and crystallinity. Furthermore, the impacts of these primary qualities on the material’s applicability for use in various fields are analysed.

Chitin is a polysaccharide found in the shells of animals such as crab and shrimp. They have also been formed from the shells of fungi [54]. Chitosan is a linear polysaccharide composed of β-(1→4)-linked D-glucosamine (deacetylated unit) and *N*-acetyl-D-glucosamine (acetylated unit) groups [55] These two are connected by β-1,4 glycosidic bonds, which assume an exceptionally vital part in the properties of the polymer [56,57]. It is a partially deacetylated chitin product, with 50–95% of the *N*-acetyl groups removed from the molecule depending on the deacetylation method used [58]. The level of deacetylation, which suggests the proportion of D-units compared with A-units, can fundamentally influence the solubility and biological activities of chitosan [58,59]. Chitosan is a white, water-insoluble, tasteless, translucent, and non-toxic solid. When reacted with polyelectrolytes and acids, it can lead to the formation of salts. Its solubility depends on factors such as temperature, molecular weight, degree of deacetylation, and anionic characteristics [60] (Rangel-Mendez et al., 2009). The capacity of the polycationic form of chitosan is a function of the presence of the free amino group and its solubility [58].

**Figure 2:**
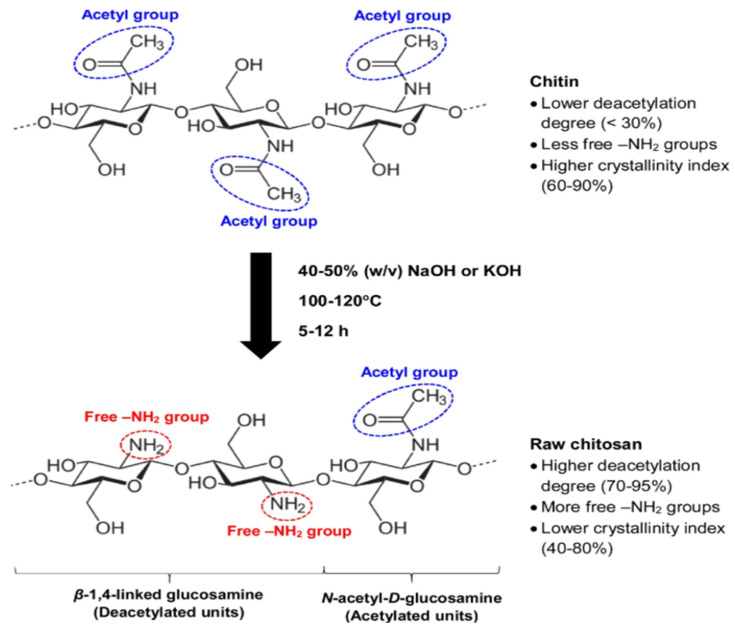
Chemical structure of chitosan and chitin after partial *N*-deacetylation of chitin to raw chitosan under alkaline conditions. Reproduced with permission from [61] Chang, 2021. Copyright (2021). Elsevier with Licence number: 5943050199935.

The degree of deacetylation (DDA) is a structural feature that distinguishes chitin and chitosan, and potentiometric or spectral analysis via FTIR and NMR are standard methods for estimating DDA [61] (Chang, 2021). Chitosan’s amine groups (Figure 2) are far more reactive than chitin’s acetamide groups. Many studies have been conducted to tailor the structure–function properties of amine groups for various applications [62] (Maia et al., 2020).

Chitosan and its derivatives have been used in environmental remediation [63], the pharmaceutical industry, medicine [64], and chemical synthesis [65]. Their wide usage can be linked to their high adsorption capacity, good biodegradability, low toxicity, and biocompatibility. The simultaneous adsorption of several heavy metal ions from wastewater has been successfully carried out by using chitosan and its derivatives [66]. Apart from heavy metals, they have been used to adsorb phenols [67], pigments [68], fluorides [69], and others.

Biomedical, pharmaceutical, biomaterials, water treatment, and hair and skincare products are among the areas of application and research. However, the applications of chitosan are limited due to its difficulty in modifying its structure and poor water solubility. The disadvantage of its application is that chitosan suffers from flaws such as low thermal stability, low specific surface area, low mechanical strength, low acid stability, etc. It possesses a lot of amino and hydroxy functional groups, which can form bonds with the heavy metal ions. These functional groups can also be modified via chemical methods to overcome limitations associated with the adsorption of pollutants [70]. Oligomers of chitosan behave in a different manner from chitosan itself. For instance, the oligomers dissolve in the basic and acidic pH range, unlike high-molecular-weight chitosan, which only dissolves in acidic medium even when the degree of acetylation is very high. Due to the solubility challenge in basic and neutral conditions, the application of this form of chitosan is limited under these conditions. This necessitates the need to synthesize derivatives of chitosan with improved solubility that could be used for various applications.

There are wide varieties in the molecular weight of chitosan; this impacts its physical and synthetic properties. Chitosan with lower molecular weight regularly has a less compact structure, bringing about more weak intramolecular hydrogen holding. This can influence its solubility and reactivity, making it more powerful for specific applications [58]. Then again, higher atomic weight chitosan will, in general, display more prominent mechanical strength and steadiness. Chitosan contains various hydroxyl and amino gatherings, and this is answerable for its gel-shaping capacity and its communications with different particles; however, its broad hydrogen security arrangement improves its utility in biomedical applications like medication conveyance frameworks and wound dressings [71].

The mechanical properties and biodegradability of chitosan are impacted by its changing level of crystallinity. The crystalline regions are normally more resistant to biodegradation, making chitosan a strong material, while the amorphous region is more prone to enzymatic activity [56,58]. The balance between the crystalline and amorphous areas is fundamental for tailoring chitosan for specific applications.

### 3.2. Sources and Formation of Chitosan

Feasible practices and upgraded use of chitosan require figuring out its sources and technique for creation. The main sources of chitosan are traced to marine organisms and, to some extent, in insects and fungi. Chitosan occurs naturally as a polysaccharide formed from chitin, usually found in the exoskeletons of exoskeletons like shrimp, crabs, and lobsters. Chitosan is an important material in different industries, including medication, farming, and food innovation, because of its biodegradability, biocompatibility, and non-poisonousness [54]. Certain insects, like beetles and subterranean insects, have exoskeletons rich in chitin, which can be processed to form chitosan [58]. Besides, a few fungi, such as Mucor and Aspergillus, likewise contain chitin in their cell walls, introducing an alternative source for chitosan extraction [54].

The extraction procedure includes gathering chitin-rich waste from fish handling ventures followed by treatment with soluble or corrosive answers to eliminate proteins. This is then trailed by demineralization by means of corrosive treatment to eliminate minerals like calcium carbonate before conclusive deacetylation into chitosan through basic or enzymatic treatment. The creation of chitosan includes the deacetylation of chitin through substances or naturally. The synthetic strategy includes treating the chitin with concentrated soluble arrangements, for example, NaOH, to eliminate the acetyl bunches coming about in chitosan [58]. In certain occasions, corrosive arrangements are utilized to upgrade the dissolvability of chitin before soluble treatment, making the deacetylation cycle more productive [54]. While organic deacetylation utilizes explicit compounds, for example, chitinases, to change over chitin into chitosan. This technique is viewed as more harmless to the ecosystem and can yield chitosan with explicit properties custom-fitted for specific applications [72]. It ought to be noticed that the decision of source and creation technique essentially impacts the quality and usefulness of chitosan. Factors like the level of deacetylation, sub-atomic weight, and immaculateness are basic for its application in different fields. Maintainable obtaining rehearses and eco-accommodating creation strategies are fundamental for limiting natural effect and upgrading the practicality of chitosan as a green material [54].

## 4. Physical and Chemical Properties of Chitosan and Its Derivatives

The degree of acetylation at C-2 determines the structure of chitin and chitosan biopolymers. Chitin is completely acetylated, whereas chitosan has amine or *N*-acetyl groups (-NHR; R = H and R = acetyl). When the level of chitin deacetylation reaches 50% or higher, the resulting biopolymer contains more glucosamine units and is referred to as chitosan. Aqueous acidic media is more soluble than chitin [73]. Chitosan is not toxic, soluble in acids, but insoluble in water. The amine groups (–NH_2_) on chitosan molecules are protonated in acidic solutions (below pH 5) and thus acquire a positive charge (–NH_3_^+^), influencing solubility, adsorption, and antimicrobial capacity [74]. According to research findings, chitosan is soluble in 10-camphorsulphonic acid, *p*-toluene sulphonic acid, and dimethylsulphoxide [74]. H_2_SO_4_ is rarely used to dissolve chitosan because it produces insoluble chitosan sulphate. Chitosan should not be dissolved in a medium containing fatty acid [75].

Chitosan stretching vibration bands appear around 3450–3400 cm^−1^ and are attributed to v(N-H) and (O-H). Stretching vibrations in 2940–2850 cm^−1^ could be attributed to the CH_3_- group in NHCOCH_3_, the CH_2_- group in CH_2_OH, and the CH_2_- group in the pyranose ring [76] (KASAAI, 2008). The vibrations at 1680–1620 cm^−1^ and 1550–1300 cm^−1^ were attributed to (C=O) in the NHCOCH_3_ group (Amide I band) and (C-N) in the Amide II band, respectively. Furthermore, the region between 1000 and 1180 cm^−1^ is typically saturated due to three distinct vibrational modes of C-C, C-O-H, and C-O-C ring vibrations [77] (Kolhe & Kannan, 2003). According to Costa et al., (2015) the viscosity and flow rate assessment of chitosan solutions made in ethanoic acid/sodium acetate and ethanoic acid/NaCl are significantly dependent on the extent of deacetylation of chitosan molecules [78].

Its functional properties determine chitosan’s chemical properties. The acetylated part could associate via hydrogen bonding and engage in hydrophobic relationships, which significantly improves the molecule’s stability, which impacts certain rigidity, strengthening its structural features. The amine and hydroxyl functional groups on chitosan are modifiable, which could enhance its available properties [75]. Chitosan’s chemical properties allow many chemical interactions with organic and inorganic species. Chitosan’s chemical properties are also linked to biological properties, including biocompatibility, biodegradability, and unique interactions with different living tissues [79,80]. Some chitosan properties, such as toughness and water absorption, were modifiable via polyvinyl acetate modification [81]. Attachment of transition metals to chitosan is primarily accomplished by coordinating metal with the -NH_2_ group of chitosan in a mole ratio of 1:1 [82].

## 5. Modification of Chitosan

Based on the capacity required, chitosan has been modified to increase the functional groups that will enhance adsorption [83]. One of the properties that was affected by the modification is solubility. Its solubility in organic acid solutions has impacts on the strength of the intramolecular hydrogen bonding, the strength of their ions, and the arrangements of the acetyl groups [84,85]. Derivatization of the functional groups and the resizing of the pore diameter under varied pH were found to be responsible for the enhanced performance of modified chitosan in acetic acid [86]. Cross-linking and grafting are two standard chemical modification processes that transform chitosan into composites.

### 5.1. Cross-Linked Chitosan

**Figure 3:**
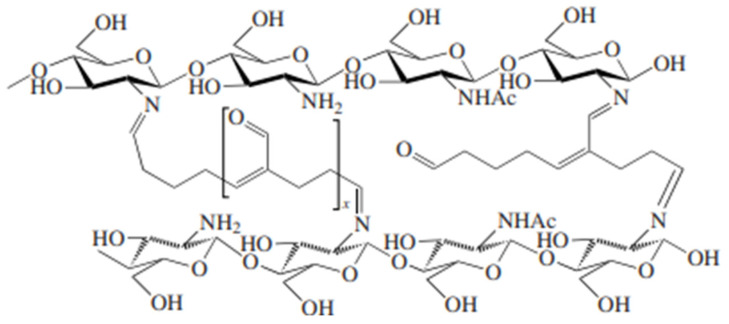
Chitosan cross-linked glutaraldehyde [89] (Kildeeva et al., 2009).

Three-dimensional network structure of polymers can be obtained from linear polymers by introducing extrainteractive bonds on the active sites of two or more polymer chains. Cross-linked chitosan is created by inducing cross-linking reactions between chitosan chains with cross-linking agents [87]. Cross-linking agents commonly used include glyoxal, formaldehyde, glutaraldehyde, epichlorohydrin, carbodiimide, boric acid, sodium trimetaphosphate, *N*-methylene bis acrylamide, polycarboxylic acid, and others [88] There are natural cross-linkers, which include transglutaminase, tyrosinase, peroxidase, laccase, sortase A, genipin, vanillin, tannic acid, and phytic acid. They work by binding to the heteroatoms on the chitosan. One glutaraldehyde molecule, for example, reacts with amino groups of the chitosan chains to form cross-linked chitosan molecules (Figure 3).

The cross-linking agent is essential because it effectively stabilizes covalent cross-linking, the formation of ionic bonds, and physical cross-linking due to hydrogen bonds or van der Waals forces. The introduction of a cross-linking agent into the chitosan structure is determined by its molecular weight, chemical structure, and the presence of active groups in chitosan [60] (Rangel-Mendez et al., 2009). The compatibility of the chitosan and cross-linking agents to produce appropriate interactions is an important factor to consider. Chitosan’s molecular structure could be cross-linked if it has a low molecular weight (typically less than 1 × 10^4^ g/mol). This form of chitosan can be cross-linked and achieve appropriate thermal, structural, and mechanical properties [60].

**Figure 4:**
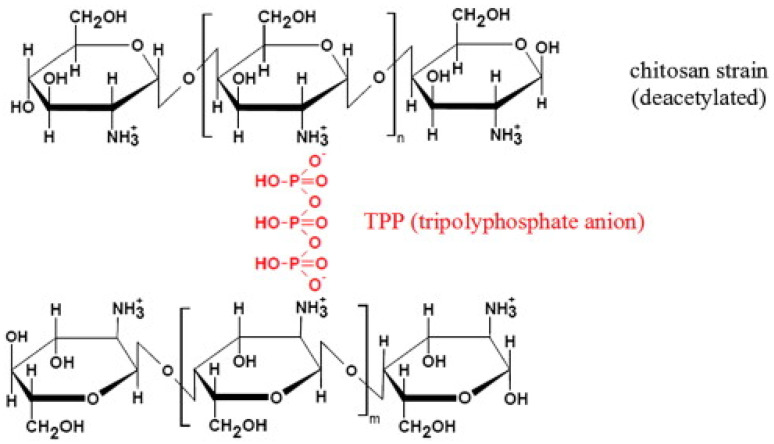
Schematic representation of ionic cross-links in chitosan modified with tripolyphosphate, TPP. Adapted with permission from [76] Gierszewska & Ostrowska-Czubenko, (2016). Copyright (2016) Elsevier. License number: 5943031419472.

Tripolyphosphate (TPP) can act as a polyanionic agent that can interact via electrostatic means with NH^+^_3_ groups in chitosan to form a stable complex. Unlike most cross-linking agents, polymer chains interact via covalent bonds (see Figure 4). Cross-linked chitosan has improved mechanical properties as well as acidic stability. Moreover, cross-linking may reduce the efficiency of adsorption because the cross-linking agent binds to OH and NH_2_, making them less accessible [90].

In a study of the adsorption of dyes using cross-linked chitosan, microbeads with small particle sizes demonstrated the highest adsorption capacity (1936 mg/g) [90] (Chiou and Li, 2002), which shows that the particle size of the cross-linked chitosan affects the adsorption capacity of the material. According to Tillet et al. (2011) [91], chitosan cross-linking can be performed at room temperature or above room temperature (intermediate temperature), which is usually around 150 °C (2011). Cross-linking agents that can react with the amine group of chitosan in an aqueous solution are used at room temperature. Enzymatic reactions and physical cross-linking of chitosan are typically carried out at room temperature. This cross-linking agent primarily uses coatings, hydrogels, protein-polysaccharide blend films, latex, and emulsions with antimicrobial or antifungal properties, and biological applications.

### 5.2. Grafted Chitosan

Graft copolymerization of chitosan, unlike cross-linked chitosan, involves polymers other than chitosan [92]. Synthetic polymers with specific properties can be introduced into chitosan molecules via graft copolymerization, improving chitosan performance and broadening its application. In a study, the chitosan-g-poly(acrylamide-acryloyloxyethyl) trimethylammonium chloride (CS-g-PAD) displayed better adsorption capacity in the treatment of wastewater than ordinary chitosan [93]. One of the compounds that has been used to form graft polymerization is phenolics. Phenolics are commonly found in wine, coffee, tea, cocoa, dry legumes, olives, cereals, vegetables, and fruits. Grafting of phenolic to chitosan is beneficial because phenolic alone has been used for different biological applications, such as in regulation of metabolic activities, anticancer agents, anti-inflammatory medications, antidiabetics, antimicrobial agents, and antioxidants [94]. Examples of phenolic compounds that have been grafted into chitosan are chlorogenic acid, syringic acid, gallic acid, sinapic acid, ferulic acid, gentisic acid, α-resorcylic acid, caffeic acid, coumaric acid, protocatechuic acid, vanillic acid, salicylic acid, and *p*-hydroxybenzoic acid [94]. The grafting of the chitosan with these phenolics is achieved by chemical coupling, enzyme catalysis, free radical mediation, and electrochemical methods [94].

### 5.3. Nanoparticles and Functionalized Form of Chitosan

Most chitosan adsorbents in powder form are submicron-sized to the micron, with little porosity and a large adsorption surface area. Furthermore, particle diffusion limits mass transfer, resulting in an adsorption rate limitation. Nano-scale adsorbents with increased specific surface area and improved performance have been developed to address these faults. Several methods for producing chitosan nanoparticles have been reported. For example, Radwan et al. (2020) used gamma radiation to create chitosan nanoparticles [95]. Qi et al. (2004) and colleagues created chitosan nanoparticles by ionic gelating chitosan with tripolyphosphate (TPP) anions [96]. Chitosan nanoparticles have better adsorptive efficiency than flaky or powdery forms, but it is difficult to recycle.

To improve chitosan solubility, the molecule’s free amino and hydroxyl groups can undergo substitution reactions to enhance their hydrophilic character. Carboxymethylation is a common strategy that produces O-carboxymethyl, *N*-carboxymethyl, and N, O-carboxymethyl chitosan [97]. The smoothness and porosity of the chitosan that has not been modified are different from those of the modified form of the chitosan. For instance, N, O-carboxymethyl chitosan has a microporous structure, while that of ordinary chitosan is non-porous and smooth. This positively affects the contact area of the modified form of the chitosan. Chitosan that has been carboxymethylated has better adsorption capacity than ordinary chitosan [97].

Quaternization is another standard method for modifying the functional groups in chitosan. Through this method, the solubility and positive charge in the material are improved [98].The primary amino group on the chitosan backbone can be directly transformed into the quaternary ammonium group. The trimethylation of chitosan’s amino groups is one form of quaternization. An alternative method for introducing quaternary ammonium salts is to react -NH_2_ and -OH groups of chitosan with quaternary ammonium reagents. The most common method is to use a linker to connect quaternary ammonium groups to the primary -NH_2_ on the chitosan’s backbone, but -OH groups can also be substituted. Reports have been of quaternized chitosan being used to remove pollutants [99]. According to Cai et al. (2010) [100], quaternized carboxymethyl chitosan flocculant synthesized by quaternization of N, O-CMC has a chemical oxygen demand removal efficiency of over 80% at pH 5. Although there have been some successful attempts, quaternization modification is not used alone to prepare chitosan adsorbents because soluble chitosan adsorbents are difficult to recycle after application.

## 6. Methods of Modifying Chitosan

### 6.1. Physical Modifications of Chitosan

Chitosan can be physically modified into various forms such as beads [66], films, membranes, and fibres to enhance its adsorption capacity. Beads are commonly prepared through the ionic gelation process using cross-linking agents like sodium tripolyphosphate (TPP) [101]. This increases the surface area and mechanical strength of the beads, making them ideal for use in fixed-bed column adsorption systems. Films and membranes are prepared by casting chitosan solutions and can be used in filtration systems for wastewater treatment [73]. These physical modifications improve chitosan’s diffusion properties and facilitate solid–liquid separation after adsorption. Chitosan nanocomposites, such as chitosan-based hydrogels, have been exploited in the removal of a wide range of organic and inorganic pollutants in wastewater [102,103,104]. Other physical modification processes include mechanical grinding, use of radiation, use of sound, use of enzymes, and use of plasma [105].

### 6.2. Chemical Modifications of Chitosan

Chemical modifications, including cross-linking and grafting, significantly enhance the adsorption performance of chitosan. Cross-linking involves the introduction of cross-linkers like glutaraldehyde or epichlorohydrin, which improves chitosan’s mechanical strength and chemical stability, particularly in acidic conditions. This modification also enhances its thermal stability and reusability [101,106]. Grafting introduces new functional groups (e.g., acrylonitrile, polymethyl methacrylate) to the chitosan backbone, increasing the density of adsorption sites and improving the material’s efficiency in capturing pollutants such as dyes and heavy metals [101,107,108].

One of the chemical modification methods involves the use of Schiff bases made from carbonyl compounds. The reaction could be used for protecting the NH_2_ group of the chitosan while the -OH group that participates in the reaction is freed by lowering the pH of the system or by adding powerful reducing agents such as sodium borohydride to the system [105]. Apart from using Schiff base, carboxymethylated functional groups are introduced to chitosan to enhance its ability to form film and improve its solubility. The reagent to be used for this purpose depends on whether the carboxymethylation will be *O*-, *N*-, *N*, *N*-, or *N*, *O*-carboxymethylation. Isopropanol, water, sodium hydroxide, and monochloroacetic acids are needed for O-carboxymethylation, while sodium cyanohydride and glyoxylic acids are needed for *N*- or *N*, *N*-carboxymethylation. Other reactions that are used to modify chitosan chemically are alkylation (using halogenated hydrocarbons at a high pH), acylation (using the derivatives of organic acids such as acid halides and anhydrides), and quaternization (introducing the derivatives of quaternary ammonium salt to chitosan) [105].

### 6.3. Impact of Modification on Adsorption Efficiency

The various modifications of chitosan, both physical and chemical, have a profound impact on its adsorption efficiency. For instance, cross-linked chitosan beads demonstrate a significantly higher adsorption capacity for heavy metals compared to non-cross-linked forms due to the enhanced availability of functional groups and improved mechanical stability [73] Similarly, grafted chitosan with side chains like acrylonitrile or polymethyl methacrylate exhibits improved adsorption efficiency by increasing the density and accessibility of active sites. These modifications also widen the pH range for adsorption, making chitosan more versatile for different environmental applications [106]. By undergoing physical and chemical modifications, chitosan becomes a highly effective and versatile material for the adsorption of a wide range of pollutants, from heavy metals to organic dyes and even pharmaceuticals. These improvements have broadened the applicability of chitosan in environmental remediation, particularly in wastewater treatment [106].

## 7. Adsorption of Organic Pollutants by Chitosan-Based Materials

Organic pollutants are among the top groups of contaminants that constitute a great threat to the availability of clean water. The most prominent among them include antibiotics, dyes, pesticides, microplastics, parabens, PAHs, and PCBs. Adsorption has been proven to be highly effective in remediating these pollutants in the environment. However, there is a need for more efficient materials with good adsorptive properties that are yet renewable, readily available, and environmentally compliant. Among such materials are chitosan-based materials.

### 7.1. Adsorption of Antibiotics

**Figure 5:**
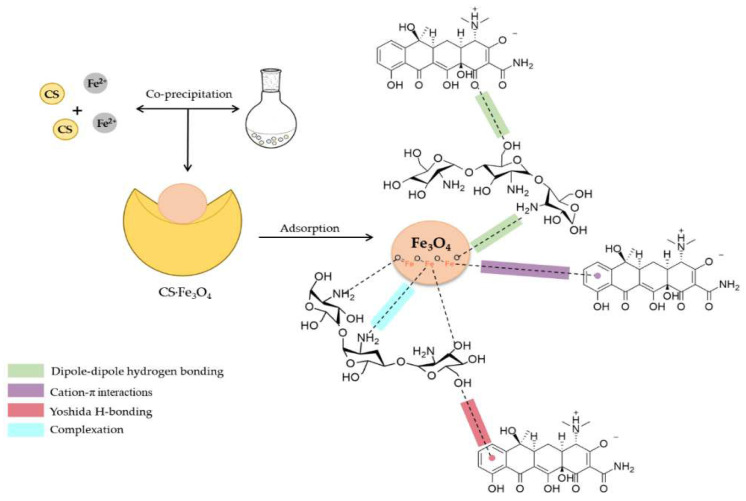
Hypothetical mechanism for the adsorption of TC onto CS.Fe_3_O_4_. Reproduced from [110]. MDPI Open access under Creative Common Agreement.

Antibiotics are part of the prominent organic pollutants of water. They are easily accumulated in water via discharge from medical environments. Antibiotics are a key class among the emerging pollutants because of the harm they cause to the ecosystem. Examples of common antibiotics include amoxicillin, norfloxacin, sulphamethoxazole, ofloxacin, tetracycline, levofloxacin, ceftriaxone, erythromycin, doxycycline, cefotaxime, and ciprofloxacin. The major mechanism of adsorption of antibiotics had been reported using chitosan-based materials to be attributed to hydrogen bonding, and electrostatic attraction (π–π and π–*n* interactions) [109]. Among the prevalent antibiotics in the environment is tetracycline (TC). The concentrations of TC in wastewater can be up to ng L^−1^ [110] Adsorbent generated from chitosan-based magnetic had been successfully exploited for the removal of TC from water. Chitosan (CS) not only served as the support for the magnetite, Fe_3_O_4_, but also displayed its adsorptive, coagulative, and chelating properties [111] The reported adsorption efficiency of the catalyst was up to 76.33% (211.21 mg g^−1^) at pH 7.0. The mechanism of adsorption was aided by the hydrogen bonds and cation–π interaction. Hence, the process is pH- and temperature-dependent, and it obeys Sips isotherms. Figure 5 shows the hypothetical mechanism suggested for the adsorption of TC via the use of the CS.Fe_3_O_4_ catalyst.

In a related study carried out by Nasiri et al. (2022), the adsorption efficiency of up to 93.07% was recorded in the use of CuCoFe_2_O_4_@Ch magnetic nanocomposite for the removal of TC from water under ideal conditions as follows: TC initial concentration of 5 mg/L, pH 3.5, adsorbent dose of 0.4 g/L at room temperature. However, the efficiency was reduced to 67% when the nanocomposite was used in real wastewater. The result was shown to follow the Freundlich isotherm, while the kinetics obeyed pseudo-second order [112] result agreed with the report of Guo et al. (2023), where the coprecipitation technique was used in the synthesis of chitosan-modified bentonite as an adsorbent for TC. Under optimal conditions of CS/bentonite ratios, dosage of adsorbent, pH, and adsorption time, the adsorption capacity obtained was 19.32 mg/g, and the adsorption fitted well with pseudo-second-order kinetics and the Freundlich isotherm [113].

**Figure 6:**
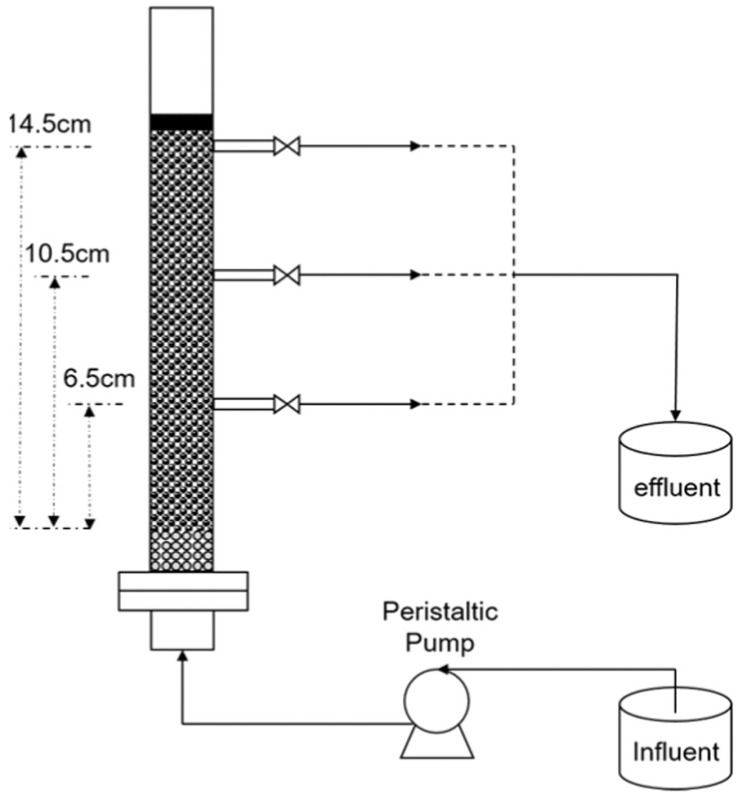
Fixed bed containing LGBW-chitosan composite for continuous adsorption of TC. Reproduced from (Yaqubi et al., 2021) [114]. Copyright (2021), Elsevier Licence Number: 5943061399299.

Development in the use of chitosan-based materials has brought about the design of a fixed bed column for continuous adsorption of TC, as shown in Figure 6. The fixed bed was packed with leached carbon black waste (LGBW) and beads made from a chitosan-carbon composite. The LGBW-chitosan composite was prepared using the instantaneous gelation technique. The flow rate of the TC-containing water was kept between 1 and 2 mL/min, while the concentration of the adsorbate (TC) was maintained at 50 mg/L. The concentration of the TC effluent was determined by UV-Vis spectrophotometer at a wavelength of 357 nm. The data obtained under the experimental conditions revealed consistency with the pseudo-first-order model for the LGBW-chitosan composites, and the adsorption process was not only supported by intraparticle interaction but was also aided by the cations present in the solution [86,114].

Moreover, the porosity of organobentonite modified with chitosan (BC) had been exploited in the removal of amoxicillin, ampicillin, and doripenem from wastewater. The temperature varied between 30 and 50 °C. According to PSO, the maximum rate of adsorption obtained and the mass of the adsorbate for the three antibiotics are presented in Table 3.

Theoretical study, a density functional theory, had been used to validate chitosan and graphene oxide as potential adsorbents for remediating the emerging pollutant, antibiotics inclusive. This was the outcome of a theoretical study carried out on the prediction of the adsorption of amoxicillin and ibuprofen on chitosan and graphene oxide materials [116]. The prediction agreed with most experimental results previously reported, and the type of intermolecular force of attraction between the adsorbate and adsorbent was suggested to be either hydrogen bond, van der Waals force, or hydrophobic interactions. This was deduced from analysis of the second-order perturbation theory. However, the interaction energies for the amoxicillin complex were found to be larger than those of ibuprofen [116].

Less-expensive hybrid beads containing chitosan had also been employed in adsorbing antibiotics from wastewater. Dutta and co-authors recently examined the adsorption of gatifloxacin from aqueous solutions using composite beads made from chitosan, walnut, and almond shell powder. Three different types of beads were prepared by varying the ratio of each of the components. The effect of contact time, adsorbent dosage, adsorbate concentration, and pH was examined. For instance, at higher pH, there was a decrease in adsorption rate due to the electrostatic repulsion between negatively charged functional groups on both beads and antibiotics. This was probably caused by the deprotonation of carbonyl groups on gatiflixacin. Thus, the optimal pH was maintained at 7. The mechanism agreed with the report by Yadav et al. (2017) [117], where it was found that the fluoroquinolone adsorption on graphene oxide was affected by changes in binding sites and surface charges of both the antibiotics and adsorbent, caused by changes in pH [118].

### 7.2. Adsorption of Dyes

Dyes will continue to be prevalent in the environment as long as some industries, such as paint, paper, textiles, and leather, are in operation. The pathetic part is that dyes are being discharged into water bodies in large masses, yet they do not easily degrade and are carcinogenic and mutagenic in nature [119]. However, chitosan, either in its native, modified, or composite form, has been reported to be used for the decolourization of dyes because of its surpassing adsorption efficiency and speed yet is easily regenerated when compared with conventional adsorbents [119,120]. The efficiency and speed of dye adsorption using chitosan have been attributed to the presence of amino groups present in chitosan. This was in support of the result obtained when cross-linked chitosan beads were employed in the adsorption of reactive black 8 [121]. In the adsorption experiment, it was discovered that the data obtained fit effectively with Freundlich, while the best-fitted kinetic model was pseudo-second order. Marotta et al. (2021) reported the use of chitosan-aerogel composite for removing methylene blue and indigo carmine from water. There was a maximum adsorption capacity of 108 mg/g and 221 mg/g for methylene blue and indigo carmine, respectively, under similar conditions [122].

### 7.3. Adsorption of Pesticides

Pesticides are among the persisting pollutants, constituting a great threat to the ecosystem because of their stable chemical structure. Hence, there is a need for a renewable and eco-friendly source for their remediation. One such material is chitosan. Dehaghi and co-authors (2014) reported the use of chitosan-zinc oxide nanoparticles (CS-ZnONPs) for the removal of permethrin, one of the prominent bioaccumulated pesticides in water bodies. The CS-ZnONPs were synthesized via a polymer-based technique. It was discovered that with just 0.5 g of the adsorbent, 99% of permethrin, with a volume of 25 mL and concentration of 0.1 mg L^−1^, was removed. One advantage of CN-ZnONPs bionanocomposite was its ability to be regenerated after being used. This was achieved by treatment with a 0.1 M solution of NaOH. Interestingly, about 56% regeneration was achieved after three cycles [123]. Ethoprophos, another renowned pesticide, had been successfully adsorbed from an aqueous solution using chitosan obtained from biopolymer waste from the marine industry. The adsorption efficiency recorded was up to 89.234% with the adsorbent dose of 0.1 g/100 mL of the aqueous solution. The energy value obtained (5.56 KJ/mol) for the Dubinin–Radushkevich isotherm mathematical expression revealed that the adsorption took place via physical means. However, among all the adsorption isotherms examined, Freundlich produced the highest correlation coefficient of 0.97 [124].

In another related study, Rissouli et al. (2017) reported the adsorption of glyphosate herbicide using chitin and chitosan. Like other reports, the adsorption process was investigated under various conditions such as pH, contact time, absorbent, and adsorbate dosage. Both Freundlich and Langmuir isotherms were also used to examine the process. The correlation values (R^2^) obtained were 0.9298 and 0.9347 for the Freundlich and Langmuir isotherms, respectively. However, a desorption experiment carried out revealed that chitosan forms a stronger force of attraction with glyphosate. Thus, it was quite difficult to recover after use. Maximum recovery for chitin was 63.76%, which is significantly greater than 9.67% observed in chitosan [125]. This may be attributed to the availability of bonding groups on chitosan rather than chitin because of the deacetylation reaction.

### 7.4. Adsorption of Microplastics

The current need for bioremediation of microplastics from the environment cannot be overemphasized because of their toxicity to the ecosystem. Microplastics have been reported to enhance bioaccumulation of pollutants in aquatic life because they possess a hydrophobic surface and a larger surface area-to-volume ratio [126]. Chitosan had been reported to be effective in the bioremediation of both nano- and microplastics in the environment. This is achievable because it has the capability of inducing the aggregation and transport of nano-plastics and micro-plastics [127]. The attraction of chitosan to the microplastics, as determined by the Derjaguin, Landau, Verwey, and Overbeek (DLVO) theory in addition to zeta potential, revealed that the attraction of the chitosan with the polystyrene (PS) nano-plastics was because of differences in charges. Interestingly, there is a need to effectively control the pH of the medium. However, the aggregation of the PS is affected by various factors such as salinity and dissolved organic matter. However, the aggregation was independent of the chitosan dosage, as the maximum aggregation of PS was achieved at a low chitosan dosage as low as 0.2 *w*/*w* [127].

The use of chitosan-based materials has also been extended to the filtration of microplastics. Risch and Adlhart [128] reported the use of chitosan/polyethylene oxide (PEO) nanofibers for the efficient filtration of microplastics from aqueous medium. The design and successful application of the nanofiber sponges constitute a breakthrough in water treatment and environmental remediation because after the hydrostatic filtration, it was discovered that 99.46% of poly(ethylene terephthalate)-MP and 99.49% of Arizona test dust had been removed by the chitosan nanofiber sponge.

### 7.5. Adsorption of PAHs, Parabens, and PCBs

PAHs, parabens, and PCBs are groups of contaminants that pose a great threat to water bodies. Thus, there is a need for their remediation via the use of low-cost, renewable, and eco-friendly materials. Such materials can be made from either native or modified chitosan. Naphthalene, a key model of PAHs, was adsorbed using chitosan beads modified with thiourea, titanium dioxide (TiO_2_), and magnetite (Fe_3_O_4_) nanoparticles. The maximum adsorption capacity of the synthesized beads (Cs-T-M-Ti) was found to be 133.690 mg/g. This value is quite high because of the large surface and ability of their constituent atoms to generate donor (D)-π–acceptor interactions. The adsorption process was found to conform with the pseudo-second-order kinetic model and Freundlich isotherm [129]. In a related study, iron-oxide-chitosan-based nanocomposites were employed in the remediation of specific common PAHs such as anthracene and phenanthrene. Different forms of iron oxide were used for the oxidative degradation of the two PAHs. The concentration of each of anthracene and phenanthrene was kept at 2 mgL^−1^, while the dosage of the nanocomposite was 20 mg. The data obtained fit well with first-order kinetics and the Langmuir isotherm, with an R^2^ of 0.99 [130]. The percentage of degradation obtained is presented in Table 4.

Moreover, the adsorptive and supportive properties of chitosan and pyrolysed chitosan were exploited in the purification of wastewater containing PAHs. Anthracene and naphthalene were adsorbed from wastewater using chitosan-derived magnet-sensitive materials prepared via a one-step co-precipitation technique. The adsorptive performance of chitosan was attributed to the functional groups (such as amine) present. However, pyrolysis carried out at temperatures of 550 °C and 800 °C increased the performance of chitosan. The same adsorbent, CS-Fe_2_O_3_ was also separately used for the adsorption of other mixtures of PAHs such as naphthalene, acenaphthylene, acenaphthene, fluorine, phenanthrene, anthracene, fluoranthene, and pyrene. It was reported that PAH molecules with higher hydrophobicity and a greater number of aromatic rings exhibited stronger sorption affinity for the adsorbent [131].

## 8. Environmental and Economic Viability of Chitosan

### 8.1. Environmental Viability of Chitosan

The fact that synthetic polymers have been discovered to damage the ecosystem calls for the consideration of other alternatives to reduce the negative consequences of plastics going forward on the planet [132,133]. Considering the biopolymers derived from natural resources is one of such options. In the short term, biopolymers cannot completely replace synthetic nondegradable polymers, but they can be employed to lessen the negative impacts of synthetic polymers. After cellulose, chitin is the second most common natural polymer. Its most significant by-product is chitosan. Chitosan is a naturally occurring polymer that is non-toxic, biodegradable, biocompatible, and antibacterial. This renewable polymer is making its way through numerous scientists’ investigations in a variety of field studies because of these qualities [134].

Furthermore, due to its numerous intriguing qualities, including biodegradability, non-toxicity, and antibacterial activity, it has a wide range of uses, from the cosmetics sector to water treatment [135]. It has been utilized in tissue engineering and medicine delivery because of its special qualities. Additionally, because of their antibacterial properties and minimal immunogenicity, chitosan nanofibers have numerous uses in the biomedical industry that will advance research and development (R & R & R&D). Its ability to be processed into a variety of shapes, including gels and sponges, makes it a perfect biopolymer [136]. Because of its antibacterial action, it is primarily researched for usage in the food industry as films that can offer a biodegradable substitute for nonbiodegradable polymers while still acting as a protective barrier. According to certain research, chitosan can be used to strengthen synthetic polymers [137,138,139].

The most popular types of shells for the synthesis of chitin and chitosan are those from shrimp and crabs. Alkaline hydrolysis is a process that can transform chitin into chitosan after it has been removed from the shell [140]. Put another way, the prawn and shrimp shell are stripped of their water, proteins, minerals, and colours to extract the chitin, which is subsequently transformed into chitosan through a deacetylation process [141]. A great substitute for synthetic polymeric materials, chitosan is seeing growth in the commercial industry. Being a less developed nation, South Africa requires new sectors and goods that may be produced from prawns and shrimp waste to favourably alter its rate of economic growth. Per hectare, production varied from 1.7 to more than 7 tons. But as industrialization increases, environmental problems and toxins are also generated, which can have a negative impact on the ecosystem [142]. Therefore, before a product is industrialized, the environmental perspective needs to be considered.

The environmental footprint of chitin production largely depends on the source material and the extraction methods employed [143,144]. The key environmental factors include:

 **1.**  **Source material utilization**

*Waste valorization:* Chitin production predominantly relies on waste products, such as shrimp and crab shells [145], contributing to waste reduction in seafood processing industries [146] (Iñiguez-Moreno et al., 2024). This circular approach significantly mitigates environmental burdens by repurposing biowaste into valuable materials [147].

*Fungal chitin:* Extraction from fungal biomass provides an alternative to marine sources, reducing dependency on seafood waste and minimizing the ecological impact of marine by-product disposal [144,148].

 **2.**  **Processing methods**

*Traditional chemical extraction:* Conventional chitin extraction involves demineralization (using acids) and deproteinization (using bases), which may produce hazardous effluents if not properly managed [149]. Efforts to improve effluent treatment and adopt less harmful reagents are ongoing.

*Enzymatic and green technologies:* Emerging methods employ enzymatic and microbial processes to extract chitin with reduced environmental impacts. These approaches consume less energy and generate minimal waste, enhancing the sustainability of chitin production [149,150].

 **3.**  **Carbon footprint and energy use**

The carbon footprint of chitin production is relatively low compared to synthetic polymers [143]. The energy demand for processing varies with the extraction technique, with enzymatic methods typically being more energy-efficient than chemical ones [151].

A comparative cost–benefit analysis underscores chitin’s advantages over conventional materials under various criteria [149,152]. The raw material, chitin, is relatively low cost due to its derivation from waste streams. In contrast, activated carbon production involves high-energy processes, increasing its overall cost. Also, the traditional chemical extraction of chitin may incur higher costs due to chemical usage and waste management, while chitin usage is narrowing this gap. Notably, chitin offers a dual benefit by repurposing biowaste and providing a biodegradable alternative to synthetic adsorbents, which can persist in the environment as pollutants. Moreover, activated carbon, although effective, often originates from non-renewable resources, adding to its environmental burden. Moreover, chitin’s biodegradability ensures minimal environmental impact at the end of its lifecycle. In contrast, some synthetic materials may require energy-intensive recycling or disposal processes (Table 5) [153].

Therefore, understanding the environmental and economic implications of chitin production is critical to fostering its adoption as a sustainable material. The significance of the current study lies in emphasizing the need for greener extraction technologies, reducing reliance on harmful chemicals, and optimizing waste management strategies. Also, utilizing waste materials for chitin production aligns with global efforts to establish circular economies and reduce environmental footprints. This cost–benefit analysis provides valuable insights for industries and policymakers, facilitating informed decisions regarding the adoption of chitin-based adsorbents over conventional materials. Lastly, chitin’s superior adsorption properties and eco-friendly nature make it a promising candidate for addressing pressing environmental challenges such as water pollution and waste management.

### 8.2. Economic Viability of Chitosan

The seafood business in South Africa is one of the sectors seeing the fastest growth, generating over R6 billion annually, or 4.7% of the country’s GDP, with a market valued at about $3 billion. As of 2017, Corporación Financiera Nacional anticipated that the market would expand by 6.45% between 2024 and 2029, reaching a value of US$2.72 billion in 2029. Of these, the shrimp sector accounts for 8%, making Ecuador one of the top exporters of shrimp globally. An Ecuadorian nation that produced 600,000 tons of shrimp in 2017 (Corporación Financiera Nacional, 2017) might and ought to think about developing a chitosan industry that uses only the shrimp shells.

Andrade reported on the feasibility of exporting shrimp wastes to China, while Berrezueta presented a business strategy for the production and marketing of chitin and chitosan in Ecuador [154,155]. Also, Chavez and Lopez offered a strategy for utilizing every component of the waste produced by the shrimp business [156]. There is not a technical report or study that has been published in the literature that covers the environmental and financial aspects of producing chitosan in Ecuador on an industrial scale. Using an anaerobic, chitinase-deficient, proteolytic enrichment culture from ground meat for deproteination and a mixed culture of LAB from bioyoghurt for decalcification, Bajaj et al. [157] investigated the scale-up of shrimp shell chitin purification in 0.25 L (F1), 10 L (F2), and 300 L (F3) fermenters. Spent fermentation liquor was repurposed for a further batch of 30 kg shrimp shells in F4 (300 L) after the deproteinization of the shrimp shells in F3. This batch removed 85.5% of the protein. The chitin that resulted had comparable diameters, F1, F2, F3, and F4. Compared to commercially available chitin and chitosan, the viscosities of chitosan generated through chitin deacetylation and chitin prepared chemically or biologically in the laboratory were significantly greater.

Numerous studies present various cost analyses and plant designs. In total, 130 tons of exoskeletons were produced from white prawns as the raw material for a chitosan production project conducted in Spain [158]. The fifth year of production and a $750 thousand investment are needed for this study to be completed. It achieves a 75% return rate and a $5 million net present value. An indication of the kind of market that might be entered is provided by the location and kind of raw materials, which generate profit for the investor. In Spain, the cost of producing 1 kg of chitosan is $14. In a different study, Gómez-Ríos et al. calculated that the manufacturing of one kilogram of chitosan would cost $10.5–12 in Colombia, accounting for a processing capacity of 230 kg/batch of dried shrimp shell [159]. It should be emphasized once more that the scaling factor is involved. Gómez et al. (2017) study uses a techno-economic method that takes into account a number of factors that virtually eliminate cost variability. Design, economic variables like the nation-risk rate, or the kind of economic investment approach, can alter our estimated cost analysis by 15% to 20%. According to research by Gómez-Ríos et al. (2017), the chitosan production factory is planned to process 5000 tons, which is equivalent to 7% of all the shrimp trash produced in Ecuador. It implies that the capacity and design of the plant may vary based on the quantity of shrimp shells considered.

**Figure 7:**
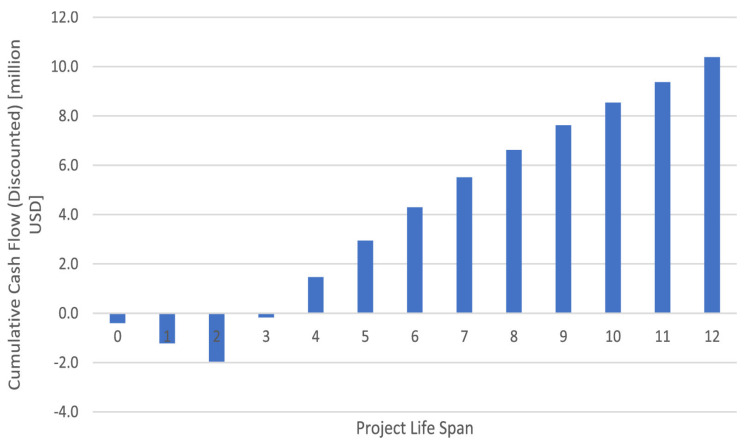
Chitosan plant cash flow ($, USD).

The anticipated cash flows from CapCost [160] are shown in Figure 1. The picture depicts the construction of the chitosan factory and the investment required in two years. Following this, the established period for chitosan manufacturing is represented as ten years. As the picture illustrates, the plant experiences a rapid return on investment, ultimately reaching a value of more than $10 million throughout its anticipated lifetime. The payback phase is reached between years 3 and 4, or around a year after chitosan production starts, as illustrated in Figure 7. Working capital, manufacturing costs, and fixed capital investment (about 1.5 million) account for the negative cumulative values from years 0–3. However, after plant building is finished, quick recovery might be observed.

## 9. Recovery and Reusability of Chitosan-Based Adsorbents

Regeneration study is very crucial before validating any adsorbent. Regeneration and recovery of an adsorbent determine its reusability. Many benefits attached to the regeneration and reusability of an adsorbent include, but are not limited to, the reduction of the cost of synthesizing fresh adsorbent, prevention of pollution that may be caused by the spent adsorbent when disposed of/released into the environment, and control of possible foul odour that may emanate from the used adsorbent when stored. This makes bioremediation feasible without creating another pollutant entirely [161].

However, regeneration of certain adsorbents depends on the nature of the desorption agent used, the toxicity of the contaminant, the technique of regeneration employed, the stability of the adsorbent, and the kind of affinity already established between the adsorbate and the adsorbent [162,163]. Chitosan-based adsorbents can be regenerated and reused after a certain number of adsorption cycles. Several techniques had been employed in the regeneration and recovery of spent chitosan-based adsorbents. Such techniques include decomposition, filtration, chemical desorption, magnetic separation, supercritical fluid desorption, thermal desorption, and advanced oxidation. However, the most suitable and commonly adopted technique for the chitosan-based adsorbent is chemical desorption, which requires the use of chemical eluents. Regeneration of chitosan-based adsorbents via chemical desorption can be carried out using various solvents (ethanol), alkaline eluents (NaOH), acidic eluents (HCl and H_2_SO_4_), chelating agents (EDTA), and salts (NaCl) [164].

Magnetic chitosan biochar (MCB) adsorbent was used for the removal of amaranth dye (a notable organic anionic dye) from the aqueous solution. The adsorbent reportedly showed a very high adsorption capacity (greater than 95%) after three cycles of adsorption and desorption [165]. The result is in agreement with that obtained in the desorption of methyl violet using magnetic chitosan/graphene oxide (Fe_3_O_4_@GO) using acetone. About 95% desorption efficiency was obtained after 4 adsorption–desorption cycles [166]. Wong et al. [167] also examined the reusability of micro-grooved chitosan (GCS) after three cycles of adsorption and desorption of methyl orange (MO). Moreover, 0.5 M NaOH was used for the desorption of MO dye from the adsorbent. It also served as the regenerative agent. The efficiency of the desorption was estimated using the following evaluation: based on the adsorbent weight and concentration of the solution, respectively. It was discovered that the adsorption of GCS reduced drastically (from 72.49 ± 0.50% to 27.86 ± 2.90% after the second cycle). In addition, 0% capacity was eventually recorded after the third cycle. The reduction in the adsorption efficiency was assumed to be attributed to the incomplete desorption of the molecules of the adsorbate. MO dyes from GCS adsorbent might have led to the blockage of some adsorption sites. The incomplete desorption may also further be attributed to the nature of electrostatic attraction between the MO molecules and GCS. This suggests that longer time would be required for the desorption process in order to obtain maximum regeneration of GCS adsorbent after two cycles of adsorption–desorption [167,168].

A regeneration study of the chitosan-reinforced graphene oxide-hydroxyapatite (CS@GO-Hap) matrix adsorbent had also been reported. It was observed that the adsorbent exhibited more than 65% regeneration ability when 0.1 M aqueous solution of NaOH was used as the regenerating agent. The dyes involved were Congo red (CR), acid red 1 (AR), and reactive red 2. The result obtained revealed that the chitosan-based adsorbent has favourable regeneration efficiency and can be reused up to six cycles without reduction in adsorption capacity [169]. Similarly, Schwarz et al. (2018) carried out an investigation of the reuse of chitosan after it has been used for the adsorption of copper sulphate, using a 10% sulphuric acid solution as the desorption medium. Insolubility of chitosan in sulphuric acid favours the desorption study. It was reported that the adsorption capacity of chitosan adsorbent, as investigated using AAS and SEM, reduced after the first cycle. However, it can be reactivated by using sodium hydroxide after the desorption process [170].

Interestingly, complete recovery of ciprofloxacin was accomplished at 30 °C with the desorption duration of 120 min when 0.1 M HCl was used as the desorbing agent with mechanical agitation of 120 rpm. Here, 100% regeneration efficiency was reported after 5 successive cycles of adsorption–desorption. The adsorbent used was chitosan/poly(acrylic acid) hydrogel, which was shown to form an electrostatic interaction with the antibiotics (Wang et al., 2019). Under the same conditions, about 85% regeneration efficiency was recorded for the desorption of enrofloxacin. Higher regeneration efficiency of chitosan-based adsorbent had been proven to be achievable because of the large number of active sites resulting in their very high stability [171].

Parlayici and Aras (2024) also examined the reusability of a chitosan-based composite used in the remediation of methylene blue from wastewater. The desorption experiment was carried out using 1% HCl as the desorption agent. Between 92% and 88% adsorption efficiency was obtained after 5 successive cycles of adsorption–desorption. The result obtained revealed that chitosan-based adsorbent possesses a promising, steady applicability without much change in its properties [172]. The same trend was observed in the desorption of humic acid from an aqueous solution using a chitosan-based adsorbent, where about 82.5% desorption efficiency was obtained. Moreover, 1 M HCl solution was also used as the desorption medium where the adsorbed epichlorohydrin was desorbed from the spent ZIF 8-doped chitosan spheres after the contact time of 120 min [173]. All these reports revealed that chitosan-based adsorbents had consistently exhibited high stability and reusability after being spent for an average of 4 cycles. However, the level of regeneration and reusability strongly depends on the factors stated earlier.

## 10. Conclusions and Future Research Directions

Chitosan, a versatile biopolymer derived from chitin, continues to attract significant attention due to its biodegradability, biocompatibility, and non-toxicity [27,174]. As research progresses, the development of chitosan-based materials has expanded into numerous innovative directions, particularly focusing on enhancing their functional properties for a large number of uses. For instance, one of the primary uses of chitosan is in water treatment, where it serves as an effective adsorbent for heavy metals and organic pollutants. Future research is directed towards enhancing its adsorption capacities through various modifications. Functionalization with nanoparticles, such as graphene oxide and carbon nanotubes, has shown promise in increasing the surface area and reactivity of chitosan composites, thereby improving their adsorption efficiency [175]. Additionally, developing chitosan-based materials with tailored pore structures can enhance their performance in capturing contaminants. More recently, studies have also discussed the varied modifications of chitosan with their enhanced absorption efficiency in comparison to natural chitosan used as a biosorbent [27,174].

Adsorption technology is pivotal for addressing a myriad of environmental and industrial challenges, particularly in water treatment, air purification, and chemical processing. A few new developments in the field indicate that adsorption techniques will become more effective, sustainable, and useful in the future. Adsorption technology, however, faces a few difficulties, such as the creation of sustainable and affordable adsorbents, material regeneration and reuse, and the transferability of laboratory results to industrial settings [13,176]. Therefore, the development of new adsorbent materials is at the forefront of adsorption technology innovation. Traditional adsorbents such as activated carbon and silica gel are being enhanced and supplemented with cutting-edge materials like graphene-based materials, metal-organic frameworks (MOFs), and nanocomposites. MOFs, for example, are highly porous materials with tunable pore sizes and surface functionalities, making them ideal for selective adsorption applications [176]. Graphene oxide and its derivatives offer high surface areas and functional groups that enhance adsorption capacities and kinetics for pollutants [177].

Functionalization of adsorbent materials to introduce specific chemical groups that can interact strongly with target contaminants is a promising trend. For instance, the incorporation of amine, thiol, or carboxyl groups can significantly enhance the adsorption capacity for heavy metals and organic pollutants [177]. Additionally, hybrid adsorbents that combine the properties of different materials—such as biochar infused with nanoparticles or polymer composites—are gaining traction. These hybrids offer synergistic benefits, including enhanced mechanical strength, increased adsorption efficiency, and improved regeneration capabilities [178,179]. For instance, the combination of biochar with inorganic materials such as clays or metals has resulted in hybrid adsorbents with enhanced removal efficiencies for heavy metals and organic pollutants [180]. These hybrids are adaptable for a range of environmental applications since they may be made to specifically target certain contaminants.

Nanotechnology has introduced nano-adsorbents with superior properties compared to their bulk counterparts. Nano-adsorbents exhibit high surface area-to-volume ratios, rapid adsorption kinetics, and enhanced reactivity. For instance, nano-zerovalent iron (nZVI) has been extensively studied for its ability to rid water of impurities like nitrates, heavy metals, and organic pollutants [181,182]. The scalability and environmental impact of these materials are areas of ongoing research, aiming to optimize their practical applications. According to a recent study, chitosan nanoparticles can be used as a possible nano-sorbent to remove harmful environmental pollutants [26]. Sustainability is becoming a critical focus in the development of adsorbent materials. Future research will emphasize creating adsorbents from renewable resources and waste materials. Bio-based adsorbents, such as those derived from agricultural waste, algae, and other biomass, present a sustainable alternative to traditional materials. These green adsorbents not only provide environmental benefits but also contribute to waste valorization and the circular economy [183].

It is a promising path to combine adsorption with other treatment technologies such as biological therapies, improved oxidation processes, and membrane filtration. Hybrid systems that combine adsorption with photocatalysis, for example, can simultaneously degrade and adsorb contaminants, providing a comprehensive solution for water purification [184]. Such integrated approaches will be crucial for meeting the increasing demands for clean water and sustainable treatment methods. Simultaneously, the use of advanced modelling and simulation techniques to understand adsorption mechanisms and predict adsorbent performance is becoming increasingly important. Computational methods, including molecular dynamics simulations and density functional theory, can provide insights into the interactions between adsorbents and contaminants at the molecular level. These tools will aid in the rational design of new adsorbent materials and optimization of adsorption processes [185].

The scope of adsorption technology is expanding to tackle emerging contaminants, such as pharmaceuticals, personal care products, and microplastics. Advanced adsorbents are being developed to effectively capture and remove these pollutants from water sources, thereby protecting public health and ecosystems. Research will continue to focus on designing adsorbents with specific functionalities tailored to these new challenges [186].

The environmental impact assessment and cost–benefit analysis reveal that chitin production, particularly through sustainable extraction methods, presents a viable alternative to traditional adsorption materials. Its low environmental footprint, cost-effectiveness, and high adsorption efficiency underscore its significance as a sustainable solution in environmental remediation and beyond. Future research and industrial efforts should focus on enhancing the scalability of green extraction technologies and expanding applications for chitin-based materials, contributing to a more sustainable future.

## Figures and Tables

**Table 1:** The difference between physisorption and chemisorption.

Physisorption	Chemisorption
Van der Waals forces and electrostatic forces, which are weak forces, hold the adsorbent to the adsorbate.	Strong covalent bonds hold the adsorbent to the adsorbate.
Comparatively, physisorption is faster than chemisorption.	Comparatively, it is slower than physisorption.
Usually involve multilayers on the surface of the adsorbents.	Usually involve a monolayer on the surface of the adsorbents.
It is rapid at a low temperature and decreases with increasing temperature.	It increases to a point and then drops at a specific point.
Accompanied with a reduction in entropy and free energy than chemisorption.	There is a reduction in entropy and free energy, but unlike physisorption.
It is easy to reverse at the critical temperature of the adsorbates or at a temperature below their critical temperature.	It can only be reversed at a very high temperature. It cannot be reversed at a temperature below the critical temperature.
Physisorption does not require activation energy to occur.	It requires activation energy to occur.
The electronic structure of the adsorbate is unaffected.	There is alteration of the electronic structure of the adsorbate due to the formation of bonds.

**Table 2:** Summary of some recent adsorption processes using chitosan-based materials as adsorbents.

Adsorbent	Adsorbate	Temperature	Adsorption Time	pH	Maximum Adsorption Capacity	Best Fitted Kinetic Model	R^2^ Value	Best Fitted Isotherm Model	References
Chitosan carbon nanotubes (CCNTs)	Amoxicillin and ciprofloxacin	-	-	-	28.885 mg.g^−1^ for amoxicillin; 40.631 mg.g^−1^ for ciprofloxacin	PFO	≥0.903	Langmuir	[46]
Chitosan biopolymer	Difenoconazole pesticide	40 OC	60 min	5.0	23.77 mg/g	PSO	0.6965	Langmuir	[47]
Chitosan beads	Amoxicillin	-	-	6.5	8.71 ± 0.6 mg/g	Simplified kinetic model	-	Langmuir	[48]
Chitosan@Polyacrylamide coated by ZIF-8	Amoxicillin and cefixime	25 OC	30 min	4.0	910 mg/g for amoxicillin and 588 mg/g for cefixime	PFO PSO And intraparticle diffusion	0.97 for amoxicillin; 0.99 for cefixime	Langmuir	[49]
Chitosan/poly (acrylic amide-co-acrylic acid) (CH/(AM-co-AA)	Amoxicillin	-	-	1.2	-	PSO	-	Freundlich	[50]
Polypyrrole-chitosan magnetic nanocomposites	Carbamazepine	-	-	-	121.95 mg/g	PSO	0.9901	Langmuir	[51]
Copper chitosan nanocomposites	Melathion (pesticide)			2.0	322.6 mg/g	PSO		Both Langmuir and Freundlich	[52,53]
2-hydroxy-1-naphthaldehyde	Pentachlorophenol (pesticide)	292–313 K		4.7–8.0	-	PSO	1.0	-	[53]

PFO is pseudo-first order; PSO is pseudo-second order.

**Table 3:** Maximum rate of adsorption obtained and the mass of the adsorbate for the three antibiotics [115].

Antibiotic	Adsorption Rate (g mg^−1^ min^−1^)	Mass of Adsorbate/Mass of BC Adsorbent at Equilibrium
Amoxicillin	6.056 × 10^−3^	53.569 mg g^−1^
Ampicillin	6.886 × 10^−3^	55.869 mg g^−1^
Doripenem	6.709 × 10^−3^	59.606 mg g^−1^

**Table 4:** Performance of different modified chitosan materials used for adsorption of anthracene and phenanthrene [130].

Nanocomposites	PAHs	% Degradation
ZnFe_2_O_4_-CS	Anthracene Phenanthrene	95 92
CuO-Fe_2_O_3_-CS	Anthracene Phenanthrene	93 90
NiFe_2_O_4_-CS	Anthracene Phenanthrene	90 88
Co_2_O_3_-Fe_3_O_4_-CS	Anthracene Phenanthrene	88 85
FeCr_2_O_4_-CS	Anthracene Phenanthrene	83 81

**Table 5:** Depicting the comparative analysis of chitin/chitosan with other adsorbent materials.

Parameter	Chitin/Chitosan	Activated Carbon	Synthetic Resins
Raw material cost	Low (waste-derived)	Medium-high (energy-intensive)	High (petrochemical-based)
Adsorption efficiency	High	High	Medium-high
Environmental impact	Minimal (biodegradable)	Moderate (non-renewable)	High (persistent waste)
Processing complexity	Moderate	High	High

## Data Availability

No new data were created.
