# Correction: Atangana et al. Adsorption of Organic Pollutants from Wastewater Using Chitosan-Based Adsorbents. *Polymers* 2025, *17*, 502

**DOI:** 10.3390/polym18020225

**Published:** 2026-01-15

**Authors:** Ernestine Atangana, Timothy Oladiran Ajiboye, Abolaji Abiodun Mafolasire, Soumya Ghosh, Bello Hakeem

**Affiliations:** 1 Centre for Environmental Management, University of the Free State, Bloemfontein 9300, South Africa; 2 Department of Chemistry, University of the Free State, Bloemfontein 9300, South Africa; 3 Industrial Unit, Chemistry Department, University of Ibadan, Ibadan 200001, Nigeria; abolajiabiodun007@yahoo.com; 4 Natural and Medical Sciences Research Center, University of Nizwa, Nizwa 616, Oman; s.ghosh@unizwa.edu.om; 5 Science Laboratory Department, Federal College of Fishery and Marine Technology, Lagos 106104, Nigeria; hakeembello34@gmail.com

There were some errors in the original publication [1].

Some text and references were missing. The corrections have been made to Sections 3.1, 3.2 and 8.2.

In Section 3.1, some texts were changed.

“Chitosan contains various hydroxyl and amino groups, and this contributes to its gel-shaping capacity and its communications with different particles; however, its broad hydrogen security arrangement improves its utility in biomedical applications like medication delivery systems and wound dressings [71].”

In Section 3.2, some texts were changed.

“Factors like the level of deacetylation, molecular weight, and purity are fundamental to its application across different fields.”

In Section 8.2, some texts were changed. The citations of references [2,3,4] were not cited, the citations have now been inserted in Section 8.2 (refs. [154–156]) and should read:

According to Statista, the seafood business in South Africa is one of the sectors seeing the fastest growth, generating over R6 billion annually.

With this correction, the order of some references has been adjusted accordingly.

“In the gazette released by the South African government, the revenue from the seafood business was projected to be US$ 2.43 billion [154,155]. A similar estimation has been reported for Ecuador where the revenue from seafood business was 4.7% of the country’s GDP, with a market valued at about $3 billion [156]. As of 2017, Corporación Financiera Nacional anticipated that the market of seafood in Ecuador would expand by 6.45% between 2024 and 2029, reaching a value of US$2.72 billion in 2029. Of these, the shrimp sector accounts for 8%, making Ecuador one of the top exporters of shrimp globally. An Ecuadorian nation that produced 600,000 tons of shrimp in 2017 [156], might and ought to think about developing a chitosan industry that uses only the shrimp shells.”

“According to research by Gómez-Ríos et al. [162], the chitosan production factory is planned to process 5000 tons, which is equivalent to 7% of all the shrimp trash produced in Ecuador. It implies that the capacity and design of the plant may vary based on the quantity of shrimp shells considered. ” 

“The anticipated cash flows from CapCost [156,163] are shown in Figure 7.”

The figure caption of Figure 7 was updated to show the source.

“**Figure 7.** Chitosan plant cash flow ($, USD) [156,163].”

The authors apologize for any inconvenience caused and state that the scientific conclusions are unaffected. This correction was approved by the Academic Editor. The original publication has also been updated.
